# County-Level Soybean Yield Prediction Using Deep CNN-LSTM Model

**DOI:** 10.3390/s19204363

**Published:** 2019-10-09

**Authors:** Jie Sun, Liping Di, Ziheng Sun, Yonglin Shen, Zulong Lai

**Affiliations:** 1 School of Geography and Information Engineering, China University of Geosciences, Wuhan 430074, China; shenyl@cug.edu.cn; 2 Center for Spatial Information Science and Systems, George Mason University, Fairfax, VA 22030, USA; zsun@gmu.edu; 3 State Key Laboratory of Resources and Environmental Information System, Institute of Geographic Sciences and Natural Resources Research, Chinese Academy of Sciences, Beijing 100101, China

**Keywords:** soybean, yield prediction, county-level, Google Earth Engine, CNN-LSTM

## Abstract

Yield prediction is of great significance for yield mapping, crop market planning, crop insurance, and harvest management. Remote sensing is becoming increasingly important in crop yield prediction. Based on remote sensing data, great progress has been made in this field by using machine learning, especially the Deep Learning (DL) method, including Convolutional Neural Network (CNN) or Long Short-Term Memory (LSTM). Recent experiments in this area suggested that CNN can explore more spatial features and LSTM has the ability to reveal phenological characteristics, which both play an important role in crop yield prediction. However, very few experiments combining these two models for crop yield prediction have been reported. In this paper, we propose a deep CNN-LSTM model for both end-of-season and in-season soybean yield prediction in CONUS at the county-level. The model was trained by crop growth variables and environment variables, which include weather data, MODIS Land Surface Temperature (LST) data, and MODIS Surface Reflectance (SR) data; historical soybean yield data were employed as labels. Based on the Google Earth Engine (GEE), all these training data were combined and transformed into histogram-based tensors for deep learning. The results of the experiment indicate that the prediction performance of the proposed CNN-LSTM model can outperform the pure CNN or LSTM model in both end-of-season and in-season. The proposed method shows great potential in improving the accuracy of yield prediction for other crops like corn, wheat, and potatoes at fine scales in the future.

## 1. Introduction

Crop yield is the most important indicator in agriculture and has numerous connections with human society. Yield prediction, one of the most challenging tasks in precision agriculture, is of great significance for yield mapping, crop market planning, crop insurance, and harvest management [1].

Remote sensing has been widely used in agricultural applications including cropland cover classification, drought stress estimation, and yield prediction by under its macro-performance and periodicity [2]. Various relevant information can be extracted from remote sensing data for yield prediction. Particularly, vegetation indices (VIs), such as the Normalized Difference Vegetation Index (NDVI), have been widely utilized [3,4,5,6]. The other indices, such as Green Leaf Area Index (GLAI) [7], Crop Water Stress Index (CWSI) [8], Normalized Difference Water Index (NDWI) [9], Green Vegetation Index (GVI), Soil-Adjusted Vegetation Index (SAVI) [10], Enhanced Vegetation Index (EVI) [11], etc., have also been used for crop production forecasting. Also, besides, meteorological variables, such as precipitation; air temperature [12,13,14]; and some soil condition data, including soil moisture, temperature, and quality, were often adopted in yield prediction as crop growth environment indicators [15].

Based on the remote sensing data, there are mainly two kinds of approaches for crop yield prediction: crop simulation and empirical statistical models [16]. Although crop simulation models are precisely simulating the physical processes in crop growth, these models can be barely applied in large spatio-temporal scales due to insufficient data. In contrast, empirical statistical models are simple and require fewer input data, and have therefore been broadly used as a common alternative to process-based models. Machine-learning algorithms, including Support Vector Machine (SVM), Decision Trees (DT), Multilayer Perception (MLP), and Restricted Boltzmann Machine (RBM) [17], can provide alternatives to traditional regression approaches and overcome many limitations. Besides, artificial neural network (ANN) was also considered as an alternative model. Traditional ANN, the multilayer perceptron model, has been applied successfully to crop yield estimation with various types of crops [18,19,20].

Recently, Deep Learning (DL) has been considered a breakthrough technology in machine learning and data mining agricultural remote sensing. Most of the DL algorithms, including Stacked Sparse Autoencoder (SSAE), Convolutional Neural Network (CNN), and Recurrent Neural Network (RNN), have been applied for yield prediction. Ma et al. proposed an SSAE for rice yield estimation using climatic and MODIS data, the result showed that the SSAE model can outperform the ANN model [21]. Kuwata and Shibasaki used a Caffe-based deep learning regression model (Gaussian Radial Basis Function) trained with remotely sensed data (satellite data, climate data, and environmental metadata) to model corn crop yield estimation at county-level [22]. Nevavuori et al. proposed a CNN model for crop yield prediction based on NDVI and RGB data acquired from UAVs, the result showed the CNN architecture performed better with RGB data than the NDVI data [23]. Yang et al. found that the CNN trained by RGB and multispectral datasets perform much better than a VI-based regression model for rice grain yield estimation at the ripening stage [24]. Chen et al. proposed a faster region-based convolutional neural network (R-CNN) to detect and count the number of flowers, mature strawberries, and immature strawberries for yield prediction [25]. Russello designed a 3D CNN architecture for crop yield prediction, the results significantly outperform competing state-of-the-art machine learning methods [26]. In addition, some researches try to integrate temporal characteristics to predict crop yield by using RNN. Jiang et al. employed the Long Short-Term Memory(LSTM), a special form of RNN method to predict corn yield with weather and soil data, the empirical results from county-level data in Iowa show promising predictive power [27]. Kulkarni et al. designed an RNN to identify optimal combinations of soil parameters and blended it with the rainfall pattern in a selected region to evolve the expectable crop yield [28]. Haider et al.developed an accurate wheat production forecasting model using the LSTM; the results verify that the model achieves satisfying performance in terms of forecasting [29]. You et al. introduced a novel method incorporating a Gaussian Process component into a CNN or LSTM; the results showed that the proposed method can outperform traditional remote sensing-based methods by 30% in terms of root-mean-squared error (RMSE) [30]. Alhnaity et al. employed the LSTM to predict yield and plant growth variation across two different scenarios—tomato yield forecasting and Ficus benjamina stem growth, in controlled greenhouse environments [31]. It is generally accepted that CNN can explore more spatial features and LSTM has the ability to reveal phenological characteristics [32]; nevertheless, to our knowledge, little attention has been devoted to comprehensively utilize the advantages of CNN and RNN for yield prediction at the county-level.

Higher accuracy and better spatial scale are two goals of crop yield prediction, they also issued a challenge for downloading, analyzing, and managing a multidecadal time series of satellite images over large areas, which is not feasible using desktop computing resources. DL has emerged together with big data technologies and high-performance computing to create new opportunities, so as to unravel, quantify, and understand the relationship between remote sensing data and crop yield at a fine scale. With the advent of Google Earth Engine (GEE), a robust connection to the Internet is now all that is required to access, manipulate, and analyze long-term global comprehensive data at various scales [33,34,35,36]. Whereas, some of the related works based on the GEE merely employ a crop statistical model for yield prediction [37,38,39], and many other DL based methods only use the GEE as a data preprocessing tool and downloaded the raw data to local drive from it, which does not take full advantage of its enormous computing power [26,30,40,41,42].

Generally, the crop yield mission always spans from the in-season to the end-of-season [43,44,45,46,47]. In the U.S., USDA provides a crop yield forecasting service, namely, Objective Yield (OY) surveys, which can provide monthly forecasts of crop yield by state-level. The OY survey field work often starts from July 25 for soybean, then the yield forecasts can be issued from Aug to the end of the season. However, the county-level soybean yield estimation cannot be issued from USDA until the next March. An early accurate county-level soybean yield prediction before that issue date is of significance for early marketing decisions and harvesting management at a fine scale. This paper proposed a deep CNN-LSTM model for both end-of-season and in-season yield prediction in CONUS at the county-level. Based on the GEE, several long-term monitored variables, including weather data, MODIS LST, and MODIS SR data, were transformed into tensors for model training; besides, historical soybean yield data was used for label and validation.

The main aims of this study are (1) to evaluate the performance of the proposed method for end-of-season crop yield prediction and (2) to explore how early a satisfied in-season crop yield prediction can be achieved. To verify the prediction power of the proposed CNN-LSTM model, two classic DL network architectures, including CNN and LSTM, were employed for comparison.

## 2. Materials and Methods

### 2.1. Study Area

**Figure 1:**
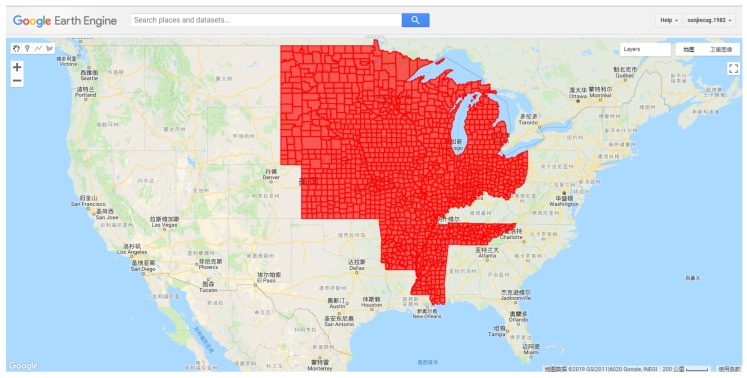
Study area in the Google Earth Engine (GEE) (red areas show selected counties).

In CONUS, the soybean was mainly planted in 31 states, in which the total cultivated area is ~33.45 million ha. In this study, 15 states of CONUS were selected: North Dakota, South Dakota, Nebraska, Minnesota, Iowa, Kansas, Missouri, Arkansas, Mississippi, Tennessee, Illinois, Indiana, Ohio, Michigan, and Wisconsin. The soybean cultivated area of these selected states is 29.69 million ha in 2015, which can account for 88.75% of the national soybean planted area [48]. Figure 1 shows the study area in the GEE.

### 2.2. Data

MODIS SR data, MODIS LST data, and Daymet weather data were taken as the influence factors. Furthermore, USDA yield data was chosen as the label data, the Cropland Data Layer (CDL) and U.S. County Boundaries data were employed as auxiliary data. Long-term and high cadence monitoring data are more likely to reveal the relationship between environmental status and the crop yield precisely. To collect training data as much as possible, the research date range was set from 2003 to 2015. Moreover, according to Usual Planting and Harvesting Dates (UPHD) of U.S. Field Crops, soybean planting and harvesting dates are usually from April to December [49]. Therefore, the date range of collected remote sensing data was narrowed from April 1st to December 31st. Except for USDA yield data, all the data can be collected and managed in the GEE. The following are the descriptions of all related data.

#### 2.2.1. USDA Yield Data

County-level soybean yield data from 2003 to 2015 were collected from the USDA [48]. The original unit of the yield is bushels per acre for each county and has been converted to kg per ha in the study. The yield data were used as labels for model training and validation.

#### 2.2.2. USDA NASS Cropland Data Layers

CDL is a crop-specific land cover data layer created annually for the continental United States using moderate resolution satellite imagery and extensive agricultural ground truth at 30m resolution [50]. The CDL is created by the USDA, National Agricultural Statistics Service (NASS), Research and Development Division, Geospatial Information Branch, Spatial Analysis Research Section. In the study, CDL data was employed as a soybean mask for two aims: the first is to mask all non-soybean pixels eliminating other interferences, and the second, to make a statistic so as to discard counties have zero soybean pixels.

#### 2.2.3. U.S. County Boundaries

The county boundaries were collected in GEE by Fusion Table format. Fusion Table is an experimental data visualization web application to gather, visualize, and share data tables.

#### 2.2.4. MODIS Surface Reflectance

Inspired by the recent successes in artificial intelligence (AI), more information can be exploited by AI compared with handcraft features, therefore raw spectral data of crop were selected instead of kinds of VI. The MOD09A1 V6 product provides an estimate of the surface spectral reflectance of Terra MODIS bands 1–7 at 500m resolution, corrected for atmospheric conditions such as gasses, aerosols, and Rayleigh scattering. For each pixel, a value is selected from all the acquisitions within the 8-day composite on the basis of high observation coverage, low view angle, the absence of clouds or cloud shadow, and aerosol loading [51].

#### 2.2.5. MODIS Land Surface Temperature

The MOD11A2 V6 product provides an average 8-day LST in a 1 km x 1 km grid. Each pixel value in MOD11A2 is a simple average of all the corresponding MOD11A1 LST pixels collected within that 8-day period. Day and night-time surface temperature bands were used for soil long-term factors [52].

#### 2.2.6. Weather Data

Daymet is a collection of gridded estimates of daily weather parameters generated by interpolation and extrapolation from daily meteorological observations. Two important weather parameters in Daymet—precipitation and vapor pressure—produced on a 1 km × 1 km gridded surface over North America were selected as climatic factors [53].

### 2.3. Method

#### 2.3.1. The Tensor Workflow in GEE

**Figure 2:**
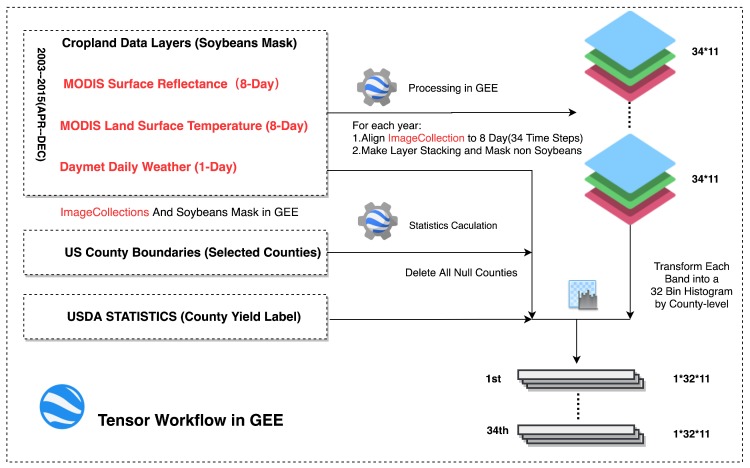
GEE-based tensor workflow.

**Figure A1:**
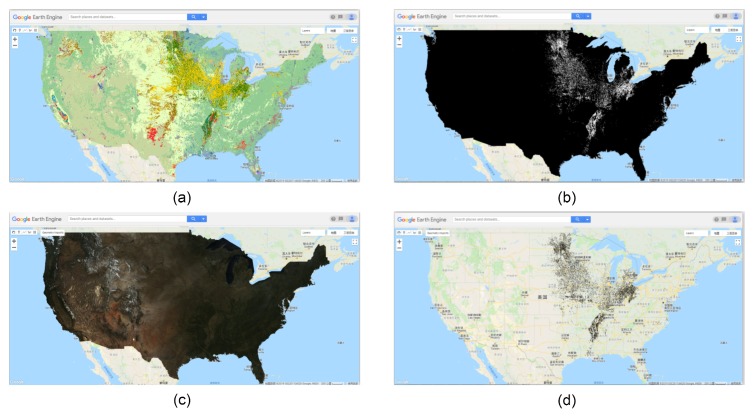
(**a**) 2011CDL. (**b**) Soybean mask. (**c**) MODIS SR(R:b1G:b4B:b3). (**d**) Masked MODIS SR.

**Figure A2:**
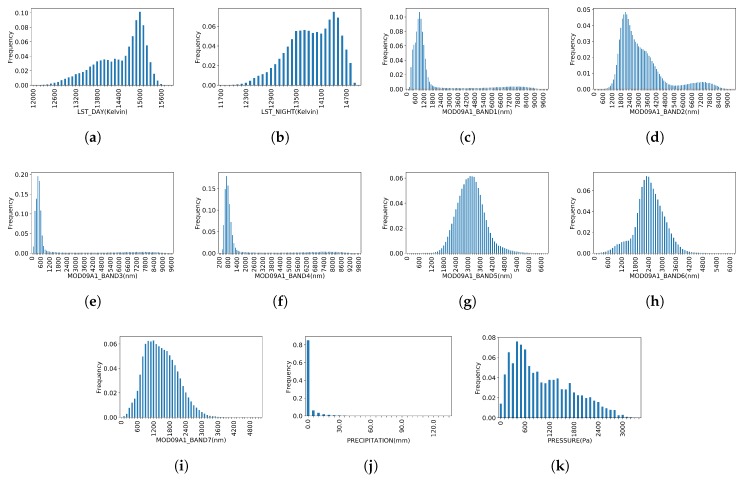
The distribution of featured bands. (**a,b**) Distribution of LST_DAY and LST_NIGHT. (**c**–**i**) Distribution of MODIS surface reflectance. (**j**) Distribution of precipitation. (**k**) Distribution of pressure.

As for a deep learning-based prediction processing, the most important initial step is wrapping the data into certain dimensional tensors for model learning. Most existing approaches preferred selecting the mean value or VI over regions as features, because these methods have low computational complexity. However, they were inclined to omit the detailed difference in a region. However, it is difficult to feed all raw remote sensing data into DL networks directly, especially for large areas on account of lacking enormous computing power. Accordingly, a histogram-based transformation was employed as a compromise, which can not only supply more information from the pixel distribution but also can take advantage of the existing cloud computing platform to improve computing efficiency. In the study, a GEE-based tensor generation workflow was proposed. Avoiding download massive data, the method can fully use the cloud computing power efficiently. Figure 2 shows the yearly tensor workflow in the GEE [54], the key steps are as follows,

1. As all the data in the GEE have been already preprocessed, ImageCollections can be made for each type of remote sensing data selected in the study according to the date range after cloud removal.
2. Crop Data Layer was employed as a soybean mask for eliminating the interference of other ground objects in all ImageCollection; the process is shown in Figure A1 of Appendix A. Besides, the counties containing no soybean pixels will be excluded.
3. MODIS SR data and MODIS LST data can be easily joined into a new ImageCollection by data system_time. Whereas, Daymet Daily weather data has a higher cadence. Therefore, they were aligned with MODIS ImageCollection after a mean values calculation at the 8-day interval in the GEE; after layer stacking, a 34×11 (time steps × features) ImageCollection for each year was prepared.
4. Before the histogram transformation, actual limits should be given. However, the theoretical limits of each band are always too wide to provide a reasonable resolution for each bin. The real distribution of each band should be calculated over the study area, which can be used as a reference for final limits. The U.S. County Boundaries data was imported as a FeatureCollection in the GEE and, combined with ImageCollections, a global statistic of each featured band was calculated covering the whole study area, then the real limits of the distribution of the pixels can be determined. Considering the capacity of GEE, all the satellite data were collected from 2003-01-01 to 2012-12-31, approximately 10 years, including 460 MOD09A1 and MOD11A2 images respectively and 521 Daymet_V3 images in the study area. The distribution of different features of soybean is presented in Figure A2 of Appendix A.
5. The GEE provides an efficient API which can transform the whole ImageCollection into a 32-bin normalized histogram by county-level. Assume *t* represents the number of time steps for each county during the season, in the study (0<t<35). Each county has an image I(t), which has *t* time steps, and each time step has m(m=11) bands with seven MODIS surface reflectance bands, two surface temperature bands two weather bands. Each band can be transformed into a histogram with n(n=32) bins. Then, each I(t) will have a histogram h(t) with the shape of t×m×n(time steps×bins×bands) as the tensor. Finally, each tensor will be assigned its corresponding county-level yield from USDA statistics; if no corresponding yield data was found in that year, the tensor will be abandoned.

#### 2.3.2. Model Architecture

**Figure 3:**
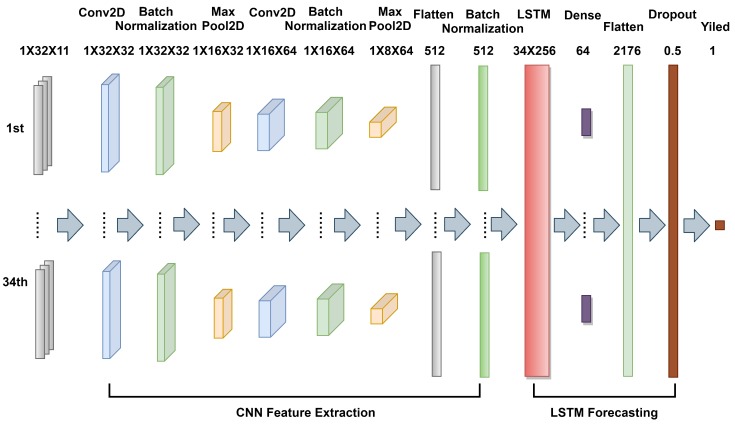
The architecture of the proposed CNN-LSTM model.

Due to the nonlinearity and complexity of the features, it is important to build a deep learning framework for yield prediction. Inspired by the success of CNN and RNN, a CNN-LSTM network was proposed in the study, which mainly consists of 2-Dimensional Convolutional neural networks (Conv2D) and LSTM networks [55]. CNN can learn the relevant features from an image at different levels similar to a human brain. An LSTM has the capability of bridging long time lags between inputs over arbitrary time intervals. The use of LSTM improves the efficiency of depicting temporal patterns at various frequencies, which is a desirable feature in the analysis of crop growing cycles with different lengths. The architecture of the proposed CNN-LSTM is shown in Figure 3. The inputs of the model are the tensors generated from the proposed GEE-based framework. The output is the predicted soybean yield. Different from traditional pure CNN or pure LSTM architectures, the proposed model mainly has two components: The first is CNN used for feature extraction, and the second is LSTM, which is used to learn the temporal features extracted by CNN. The CNN starts from two Conv2D layers with a kernel size of 1×2, the first Conv2D has 32 filters and the second has 64 counterparts. Feature maps are first followed by a batch normalization layer and then followed by a 2-dimensional max-pooling layer with 1×2 kernel. This improves generalization, robustness to small distortions, and also reduces dimensionality. Note that batch normalization is employed after each convolutional layer, which is another method to regularize a CNN. In addition to providing a regularizing effect, batch normalization also gives CNN resistance to the vanishing gradient during training, which can decrease training time and result in better performance [56]. By the TimeDistributed wrapper, the two stacked Conv2D layers are applied to every temporal slice of the inputs for feature extraction. Then, each output is flattened and batch normalized successively before they are fed into an LSTM layer. There is only one LSTM layer in the LSTM part. The neurons’ number of the LSTM is set to 256, which is followed by a dense layer with 64 neurons. After that, all temporal output is flattened into a long vector, which is sent into a Dropout layer with 0.5 dropout probability; the dropout layer can randomly turn off a percentage of neurons during training, which can help prevent groups of neurons from all overfitting to themselves. Finally, a one neuron dense layer is used to output predicted yield. The activation function of the model is ReLU (Rectified Linear Units), because it can avoid and rectify the vanishing gradient problem; the formula is shown in Equation (1). Besides, ReLU is less computationally expensive than tanh and sigmoid as it involves simpler mathematical operations.

(1) ReLU=Max(0,x)

Continual training might improve the accuracy on a dataset, but at a certain point it starts to reduce the model’s accuracy on data not yet seen by the model. To improve real-world performance, early stopping was employed for reducing the generalization error of your deep learning system. The training will end when a monitored “val_loss” quantity has stopped improving after 10 epochs consecutively. There are 100 training epochs with a batch size of 16 and gradient descent on top of the adaptive momentum (ADAM) optimizer.

#### 2.3.3. End-of-Season and in-Season Yield Prediction

The end-of-season and in-season yield predictions are studied herein. Generally, the model was trained by all the data before the prediction time node; additionally, to keep the impartiality of performance evaluation, a certain fraction (0.2) of the training data was randomly set apart for validation. Such a setting can make the most of previous data for a stable model for yield prediction. For example, if we would like to make an end-of-season prediction in 2014, the data from 2003 to 2013 is used as training data, and in each year, the training data has to cover the whole season from April to Dec and the shape of tensor per county is 34×32×11( time steps×bins×bands ); moreover, if we would like to make an in-season prediction on JUN 2, 2015, we employ training data from 2003 to 2014, and in each year the training data only range from the beginning of season to JUN 2, the shape of tensor per county becomes 8×32×11( time steps×bins×bands ). The difference between the two kinds of prediction mainly manifest in prediction time and how many time steps data will be used in the tensors. To find out how early a satisfied in-season yield prediction can be achieved, first, six time nodes, including the 8th, 12th, 16th, 20th, 24th, and 28th time steps corresponding to JUN-2, JUL-4, AUG-5, SEP-14, OCT-16, and NOV-17 in the season timeline, respectively, were selected for evaluating the potential of early yield prediction. In addition to the above time nodes, more dense time nodes could also be added in the in-season prediction if necessary.

### 2.4. Evaluation

Based on the proposed deep CNN-LSTM model, the performance of all end-of-season and in-season soybean yield predictions were evaluated. As it was demonstrated in several studies that the DL method can outperform traditional machine learning methods in crop yield prediction [26,30,57], we only focused on two classic DL network architectures—CNN and LSTM—as the baseline for comparison. Each architecture consisted of the proposed CNN-LSTM split into its main components for the same prediction task. To avoid estimation bias, the evaluation was performed from 2011 to 2015; each year has a different training data that can output a different model. Based on these models, the yield from 2011 to 2015 can be predicted. Then, compared with the observed yield, the performance of the prediction can be evaluated year-by-year as well as the 5 years’ overall evaluation. The process is similar to Leave-One-Out Cross-Validation. Some metrics, such as Root-Mean-Squared Error (RMSE) and Percent Error (PE) were selected. Formulas of RMSE and PE are presented in Equations (2) and (3), where $y_{i}$ is the predicted value, ${\hat{y}}_{i}$ is the observed value, and *n* is the number of samples. Besides, the $R^{2}$ between the observed and the predicted yield was also used, to evaluate how well the predicted values can reconstruct the spatial variations of observed yield.

(2) $$RMSE=\sqrt{\frac{\sum_{i=1}^{n}{\left(y_{i}-{\hat{y}}_{i}\right)}^{2}}{n}}$$

(3) $$PE=\frac{\left|y_{i}-{\hat{y}}_{i}\right|}{{\hat{y}}_{i}}\cdot 100\%$$

Finally, the feature importance was also evaluated. It is significant to evaluate the feature importance, which can help us understand the process of DL. Permutation Feature Importance (PFI) is a commonly used method to evaluate the importance of the input variables [58]. However, there is no universal PFI tool in a DNN, except the sequential model. The features of the study mainly divided into two types: MODIS SR, representing the crop growing status, and the other is MODIS LST and weather data, as the supplement to MODIS SR, they describe the crop growing environment. Inspired by PFI, we try to drop either type of feature, then, train the network for each of the cases and evaluate the prediction accuracy. An important feature will provide more benefit to overall prediction accuracy.

## 3. Results and Discussion

The experiment was performed under the following configuration; CPU:Inter i5-6600k 3.5G, RAM: 16GB, Disk Storage: 2T, and Software:Keras 2.2. The yield prediction mission in CONUS can be completed in one day. It is believed that the method will be more efficient on GPU.

### 3.1. Tensor Generation

According to the distribution of features in Figure A2 of Appendix A, as shown in Table 1, the new range of MOD09A1 is from 1 to 5000, differing from the original range (−100 to 16,000), the range of MOD11A2 is changed from 12,400 to 15,600, differing from the original range (−1.5 to 340,573), the range of precipitation is changed from 0 to 35 instead of the original range(0–200), and the range of pressure is changed from 0 to 3200 instead of the original range (0–10,000). Based on the new limits, the pixels of each band will be allocated into 32 bins. Given a fixed number of bins, the wider limits the wider bin width. However, with a narrower bin width, more detailed information can be represented. The original limits are theoretic values, which are often wider than the actual limits of the distribution. Therefore, it is suggested that the actual distribution limits of each band should be determined to make the histograms more discriminating.

**Figure A3:**
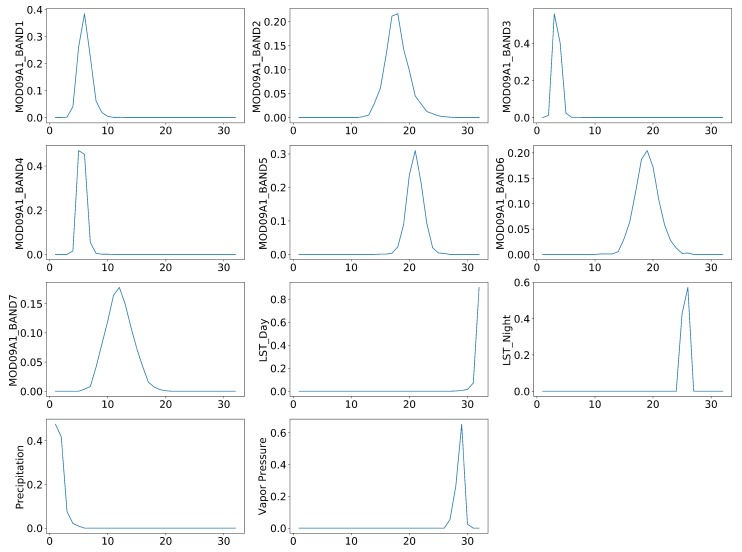
One time-step of tensors for Marion in Kansas, Aug 21st 2011.

Figure A3 of Appendix A shows the histograms of the 18th time step for the Marion (a county in Kansas) tensor in 2011. There are 11 histograms corresponding to 11 bands. Each histogram depicts the pixel distribution in 32 bins. It is expected that the deep learning networks can find out the relationship between these features and yield by its powerful learning ability, given enough training data.

### 3.2. Model Evaluation

#### 3.2.1. End-of-Season Yield Prediction

Table 2 shows the performance of the end-of-season yield prediction based on the different models including the proposed CNN-LSTM model, CNN, and the LSTM. The first five rows are predictions performances of each year from 2011 to 2015; the evaluation was performed between predicted yield and observed yield and measured in RMSE(unit:kg/ha), and the last row is an average RMSE for above five years. The result shows that the proposed deep CNN-LSTM model has the advantage of yield prediction in each year, except in 2012, and the average RMSE of the CNN-LSTM has a ~8.24% and ~9.26% reduction of RMSE from the CNN and LSTM, respectively, which indicates the proposed deep CNN-LSTM can outperform CNN or LSTM in end-of-season yield prediction.

**Figure 4:**
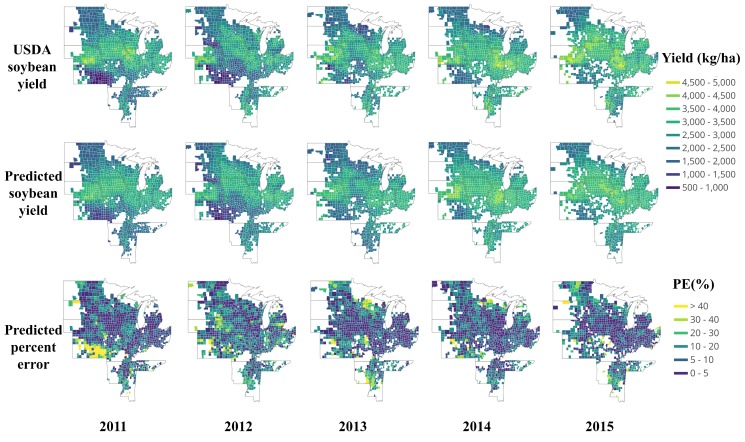
Map of USDA soybean yield, predicted soybean yield, and percent error (PE) from 2011 to 2015.

Figure 4 shows a detailed comparison of the yield distribution map between the USDA yield data and the predicted yield at the county-level. The first row is USDA soybean yield data from 2011 to 2015, and the middle row is the corresponding end-of-season predicted yield based on the proposed deep CNN-LSTM model. The dark color means low yield, and vice versa.

**Figure 5:**
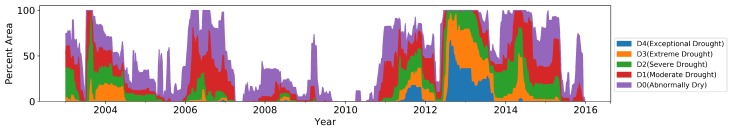
Time series for drought monitor of Kansas.

Generally, there is high consistency between the predicted yield and USDA result. Across the years, higher yield is concentrated mainly on Nebraska, Illinois, Iowa, Ohio, Michigan, and Mississippi, whereas the lower yields are typically found in Northern Dakota, Kansas, and northern Wisconsin. To further reveal the performance, based on Equation (3); the prediction percent error maps are also presented in the third row of Figure 4. Most of the prediction percent error is less than 10%, or even less than 5%; whereas, some extremely high prediction errors happened mostly in Southern Kansas 2011, showing a bright color. All of these counties share remarkable yield reduction. The yield reduction may attribute to many factors, including weather, soil quality, fertilization conditions, irrigation, disease, and pests. The reason for this may be related to a severe drought: Rippey [59] showed that the soybeans, somewhat more drought tolerant due to their ability to “shut down” during hot, dry spells and reproduce when cooler, wetter weather returns, experienced a nine percent yield reduction in 2012 drought of U.S. However, we found the severe drought begun in 2011 in Kansas. Figure 5 is a time series for drought monitor of Kansas from 2003 to 2015 [60]; an unusual drought happened from 2011, moreover, the drought level of Southern Kansas was D4 (Exceptional Drought), which lasted from 2011 to the end of 2013. Consequently, the drought may be one of the important factors for the large reduction of soybean yield. The reason for the poor performance of the model in Southern Kansas 2011 shown in Figure 4 is mainly due to lack of training samples. There is hardly a so long-lasting exceptional drought before 2011. Therefore, the situation is an exception for the model, which was not able to learn the causes that led to such a big difference. However, under even worse weather conditions in 2012, the model performed much better in Southern Kansas, shown in Figure 4 2012. It illustrates that the model was improved when data of 2011 was integrated into the training data. It can be concluded that extreme weather record may cause an exceptional prediction result that year and is valuable for future prediction. An increase of extreme and uncertain events is characteristic of the most recent climate scenarios, which can help DL networks learn various cases and become more universal [61].

**Figure 6:**
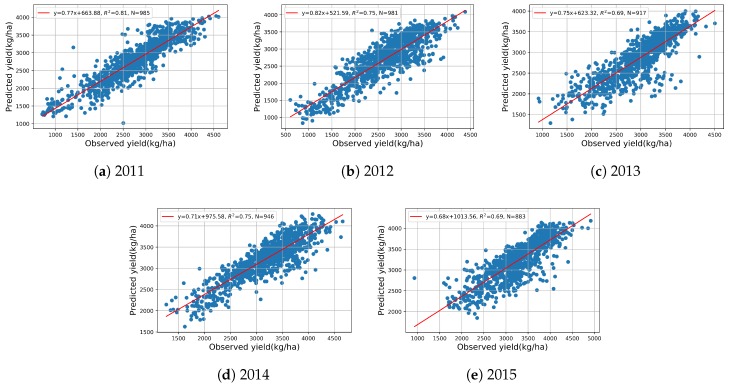
Scatter plots of end-of-season predicted vs. observed yield from 2011 to 2015.

To complement the RMSE results, the $R^{2}$ between the predicted yield and observed yield are also shown in the scatter plots in Figure 6, giving better understanding of the performance of the proposed method. From 2011 to 2015, the $R^{2}$ show the end-of-season predicted yield can explain 81%, 75%, 69%, 75%, and 69% of the variance in the observed yield, which also confirms the validity of the proposed model for end-of-season yield prediction.

#### 3.2.2. In-Season Yield Prediction

**Figure 7:**
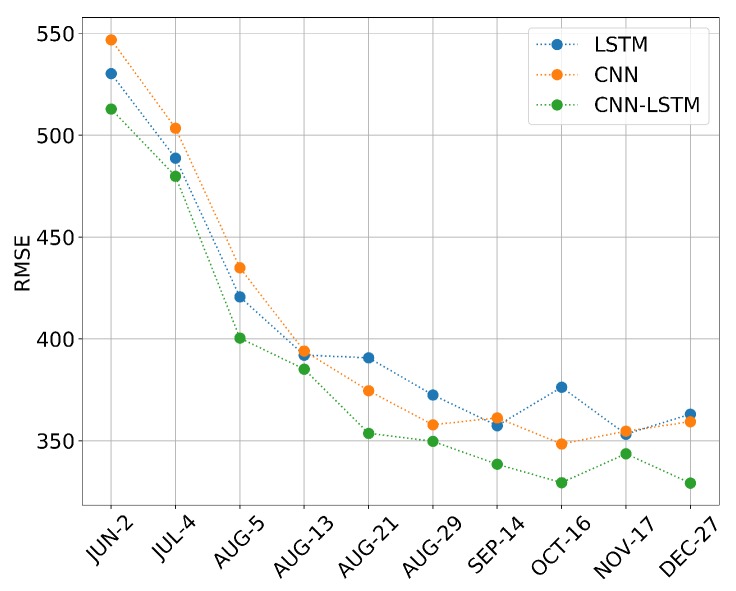
The averaged model performance measured in RMSE from 2011 to 2015.

Accurate early yield prediction is essential for market pricing, planning labor, transport, as well as harvest organization. Table 3, Table 4 and Table 5 show the performances of different models for yield prediction in early months during the soybean growing season of each year, the evaluation is measured in RMSE. The results of the three models consistently lack information for training; all the models do not perform well in the early months, such as JUN and JUL, as there is not enough information on crop growth or environment. Over time, more information was integrated into training data, and the model performance was improved gradually. Note that the RMSE of 2012 is usually relatively high than other years; this is because 2012 was a particularly dry year, and most counties in the U.S. experienced a decrease in soybean yield [59], shown in Figure 4. It seems that DL model works poorly for exceptional cases. In addition, a further comparison which averages the RMSE of all five years for each model is shown in Figure 7, the result shows that the prediction performance of all the models are improved sharply from JUN to SEP (CNN RMSE:546.75-361.14, LSTM RMSE:529.94-357.10, CNN-LSTM RMSE:513.12-338.27). All the models can achieve their best results after SEP. The best result of LSTM is in NOV (RMSE = 353.07), the best result of CNN is in OCT (RMSE = 348.36), as well as the CNN-LSTM (RMSE = 329.53). After SEP, the performance shows a small fluctuation, which may be caused by early harvesting in some counties. In short, The CNN-LSTM can outperform the other models at any time node, which proves the superiority of the proposed model for in-season yield prediction.

However, it must also be mentioned that the soybean harvesting time varies from state to state, and some states start harvesting from early OCT. Therefore, there is still an urgent need to know whether a satisfying in-season yield prediction can be achieved earlier for general instruction. As shown in Figure 7, there is a big gap between AUG and SEP, and the accuracy curve becomes stable after SEP. We wonder if we can obtain a comparable prediction result before SEP. Thus, three more time nodes were also tested between AUG and SEP. The 13th, 21st, and 29th of AUG were added in terms of the 8-day interval. At each new time node, in-season yield predictions were performed based on the three models; the performance is also shown in Table 3, Table 4 and Table 5 and averaged in Figure 7. The results show that the proposed CNN-LSTM model can still outperform the other models in each new time point. It is important to highlight that the CNN and LSTM models can obtain their best prediction results in OCT (RMSE = 348.36) and NOV (RMSE = 353.07); in contrast, their best prediction results can be nearly achieved on AUG 21st (RMSE = 353.74) by the proposed CNN-LSTM model, which can win a long time in advance compared with CNN or LSTM and only have a small increment by 7% in RMSE compared with the end-of-season prediction.

**Figure 8:**
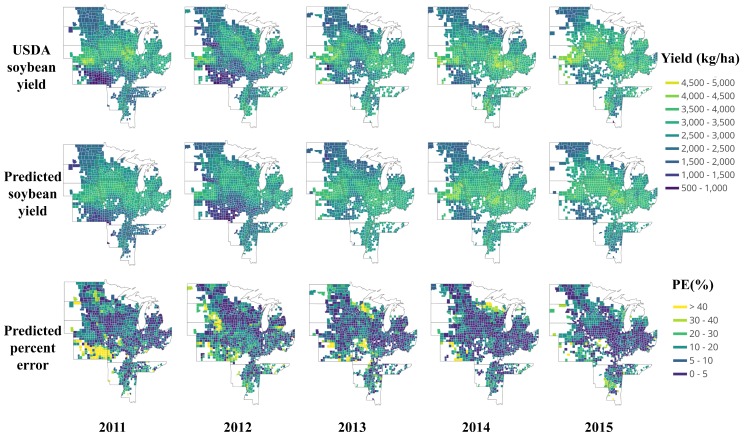
Map of USDA soybean yield, CNN-LSTM in-season predicted soybean yield and PE from 2011 to 2015.

**Figure 9:**
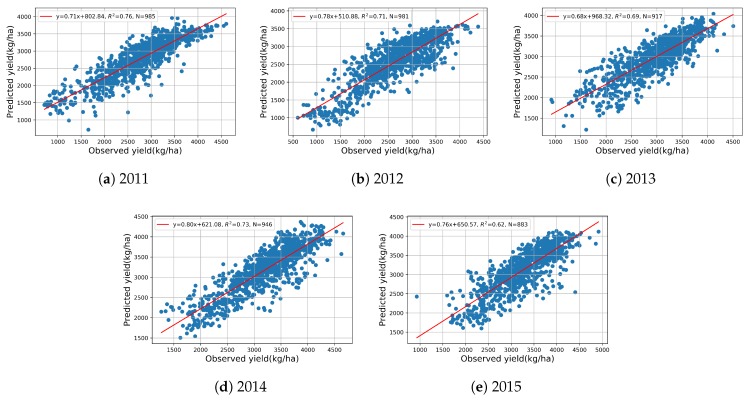
CNN-LSTM in-season predicted vs. observed soybean yield on AUG 21st from 2011 to 2015.

To further investigate the feasibility and performance of making an early yield prediction on AUG 21st by the proposed CNN-LSTM, the maps of yield distribution and prediction percent error are shown in Figure 8. Compared with the end-of-season prediction results in Figure 4, there is a little difference in the distribution of PE. Most of the in-season prediction results are consistent with the end-of-season prediction results, generally. To gain more insight, Figure 9 plots the predicted yield vs. observed yield. From 2011 to 2015, the in-season predicted yield can, respectively, explain 76%, 71%, 69%, 73%, and 62% of the variance in the observed yield of each year; these results are equivalent to the results of the end-of-season prediction in Figure 4.

**Figure 10:**
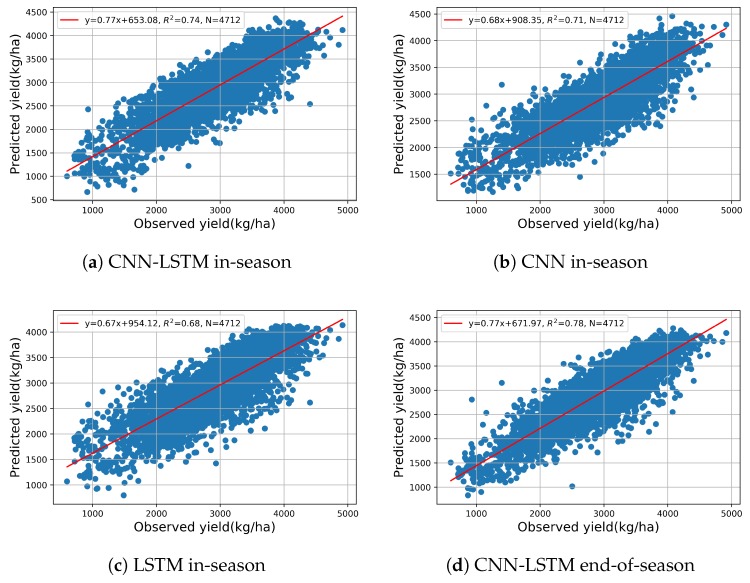
Scatter plots of five-year in-season or end-of-season prediction by different models (**a**) CNN-LSTM in-season. (**b**) CNN in-season. (**c**) LSTM in-season. (**d**) CNN-LSTM end-of-season.

**Figure A4:**
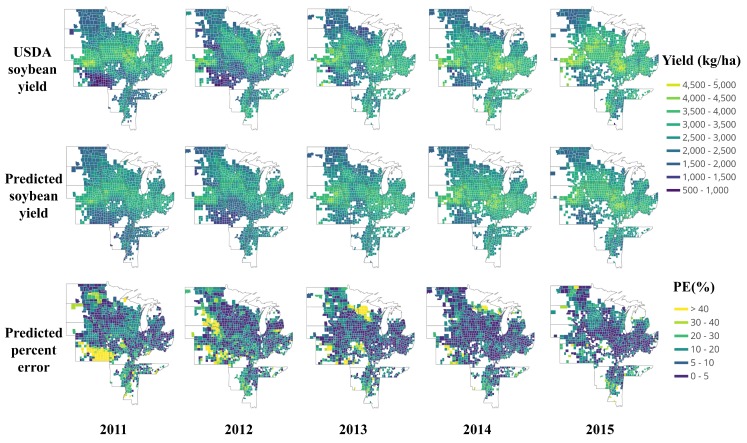
Map of USDA soybean yield, convolutional neural network (CNN) in-season predicted soybean yield and PE from 2011 to 2015.

**Figure A5:**
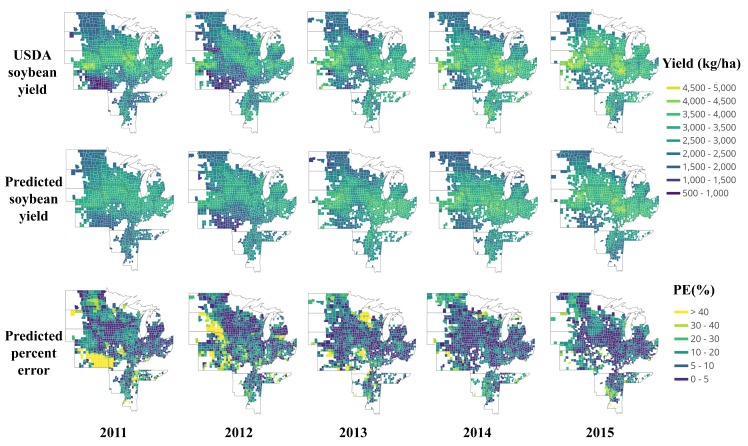
Map of USDA soybean yield, LSTM in-season predicted soybean yield and PE from 2011 to 2015.

Moreover, Figure 10 shows an overall comparison, in the same way, of the in-season predicted yield and end-of-season predicted yields of all five years, which is compared with the observed yield; $R^{2}$ illustrates that all the in-season predicted yield can explain ~74% of the variance in the observed yield, which is comparable to the value of 78% in the end-of-season prediction. On the basis of these results, we concluded that compared with CNN or LSTM, the proposed CNN-LSTM model has a better performance for in-season soybean yield prediction at county-level, and, based on the model, an accurate early soybean yield prediction can be made on AUG 21st, which would benefit farmers’ productivity and pricing in future. In addition, as the baseline, the in-season prediction results of CNN and LSTM are also shown in Figure A4 and Figure A5 of Appendix A. The models show a consistency that poor performances always happen in Kansas (2011), South Dakota (2012), and Wisconsin (2013), which is similar to the PE distribution of CNN-LSTM in Figure 8. Additionally, as shown in Figure 10, the five-year prediction scatter plots also prove that the performance of CNN-LSTM ($R^{2}=0.74$) is better than CNN ($R^{2}=0.71$) or LSTM ($R^{2}=0.68$).

#### 3.2.3. Feature Importance Analysis

**Figure 11:**
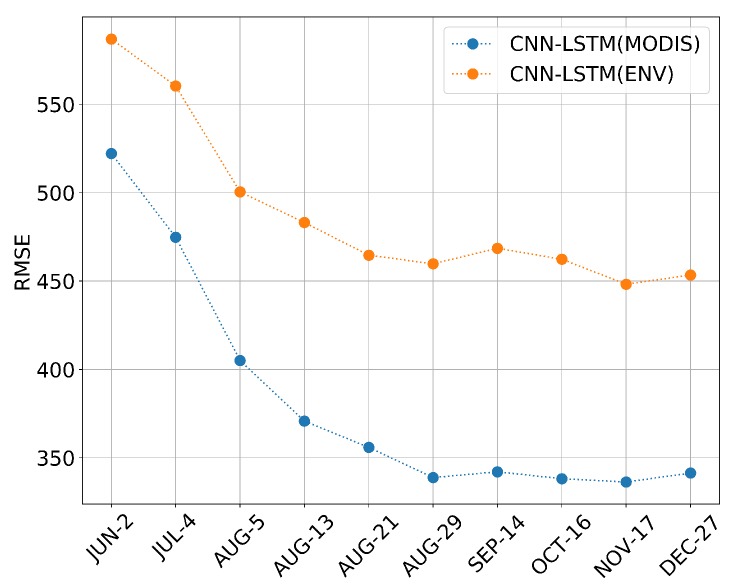
Performance of different type of features.

Figure 11 shows the averaged performance of different models. CNN-LSTM (MODIS) is the CNN-LSTM model only trained on MODIS surface reflectance, and the CNN-LSTM (ENV) is only trained on environmental variables. We are surprised that the performance of CNN-LSTM (MODIS) can outperform CNN-LSTM (ENV) at each time step. The CNN-LSTM-ENV model delivers relatively poor accuracy. The comparison suggests that, based on our framework, the MODIS surface reflectance is more important than environmental features in soybean yield prediction. There are several possible reasons: First, the impact of external factors on yield is extremely complex. These external factors include weather, soil quality, fertilization conditions, Irrigation, disease and pests, etc. It may be insufficient to make a prediction only using LST, precipitation, and pressure. Second, these factors usually have a complicated interaction with each other during the growing season. However, the impact of all these factors can be demonstrated by crop growing status finally; in other words, the environment variables are indirect monitoring values while the growing status variables are direct monitoring values. This is consistent with the conclusion of a previous study: Saeed et al. show NDVI played the most important role in wheat yield prediction compared with other environment variables in Random Forest [62].

## 4. Conclusions

Accurate early yield prediction is of great significance for crop market planning, crop insurance, and harvest management. In this paper, a GEE-based CNN-LSTM model was proposed for both in-season and end-of-season soybean yield prediction by county-level in CONUS. From 2011 to 2015, the results demonstrate for the first time evidence that (1) compared with the CNN or LSTM, the prediction performance of the proposed CNN-LSTM model was proven to be the best. Based on the proposed method, the end-of-season yield prediction can obtain high accuracy with RMSE = 329.53 averaged from 2011 to 2015 and $R^{2}=0.78$ for five years together. (2) An early prediction on AUG 21st can achieve a satisfying result with RMSE = 353.74 and $R^{2}=0.74$, which is comparable to end-of-season result but can win a long time before USDA issue data. (3) The method is highly efficient, as it can benefit from the great computing power of GEE and a dimension reduction method. (4) MODIS surface reflectance played a more important role in the method than environmental features.

However, as a preliminary attempt to investigate a U.S. county-Level soybean yield prediction using CNN-LSTM, a few improvements may be taken into consideration in future work. First, using only weather and LST data may be insufficient for yield prediction, and more features could be added to the training data such as soil moisture, soil quality, transpiration, and irrigation situation, which makes the model more comprehensive. Second, although the proposed method employs a histogram-based tensor transformation that can fuse different remote sensing data into a composite, combining multisource data with different resolution and cadence for feature extraction remains challenging, for example, some data is monthly or yearly while some of the data may be constant. To accommodate the data, some optimization should be adopted on the model architecture. Third, the resolution of tensors depends on the bin number, different bin numbers, such as 64, 128, or higher, will be tested for performance comparison. This method can offer exciting opportunities for other kinds of early crop yield predictions at larger scales in the future.

## Figures and Tables

**Table 1:** The actual data range of features.

Feature	Original Min	Original Max	New Min	New Max
MOD09A1	−100	16,000	1	5000
MOD11A2	7500	65,535	12,400	15,600
PRECIPITATION	0	200	0	35
PRESSURE	0	10,000	0	3200

**Table 2:** Model performance of end-of-season yield prediction measured in root-mean-squared error (RMSE).

Year	CNN	LSTM	CNN-LSTM
2011	337.60	372.57	312.72
2012	345.67	384.67	349.03
2013	357.10	359.12	338.27
2014	351.72	357.10	307.34
2015	404.85	341.63	338.94
Avg	359.12	363.15	329.53

**Table 3:** Model performance of the convolutional neural network (CNN) measured in RMSE, 2011–2015.

Year	JUN-2	JUL-4	AUG-5	AUG-13	AUG-21	AUG-29	SEP-14	OCT-16	NOV-17	DEC-27
2011	558.85	526.57	395.44	434.44	408.89	355.76	347.01	346.34	334.91	337.60
2012	599.21	560.20	471.43	371.90	386.69	370.55	350.38	353.74	363.15	345.67
2013	507.74	455.29	453.94	397.45	355.08	351.72	342.31	321.46	341.63	357.10
2014	504.38	480.17	412.92	381.98	349.03	346.34	343.65	319.44	323.48	351.72
2015	563.56	494.97	440.49	384.00	373.24	363.83	423.68	400.82	410.90	404.85

**Table 4:** Model performance of the long short-term memory (LSTM) measured in RMSE, 2011–2015.

Year	JUN-2	JUL-4	AUG-5	AUG-13	AUG-21	AUG-29	SEP-14	OCT-16	NOV-17	DEC-27
2011	538.68	497.66	405.52	411.58	412.92	393.42	379.97	391.40	375.93	372.57
2012	618.04	594.50	509.09	442.51	418.30	412.92	374.59	415.61	375.93	384.67
2013	524.56	429.06	390.73	386.02	367.19	340.29	332.89	373.91	338.94	359.12
2014	468.74	430.41	365.17	361.81	357.77	322.80	357.77	322.13	340.29	357.10
2015	501.02	492.28	433.10	357.77	396.78	393.42	341.63	377.95	334.24	341.63

**Table 5:** Model performance of the CNN-LSTM measured in RMSE, 2011–2015.

Year	JUN-2	JUL-4	AUG-5	AUG-13	AUG-21	AUG-29	SEP-14	OCT-16	NOV-17	DEC-27
2011	511.11	484.21	388.71	396.11	351.72	340.96	342.31	348.36	335.58	312.72
2012	597.86	574.99	449.91	435.11	377.28	359.79	343.65	353.74	347.01	349.03
2013	496.98	445.87	385.35	358.45	337.60	347.01	320.79	305.32	316.75	338.27
2014	464.03	435.11	377.95	343.65	321.46	311.37	305.32	298.59	305.99	307.34
2015	494.29	459.32	400.14	393.42	379.97	389.38	379.97	340.96	412.92	338.94
